# Dietary Acid Load, Serum Polychlorinated Biphenyl Levels, and Mortality Following Breast Cancer in the Long Island Breast Cancer Study Project

**DOI:** 10.3390/ijerph19010374

**Published:** 2021-12-30

**Authors:** Briana N. C. Chronister, Tianying Wu, Regina M. Santella, Alfred I. Neugut, Mary S. Wolff, Jia Chen, Susan L. Teitelbaum, Humberto Parada

**Affiliations:** 1 Herbert Wertheim School of Public Health, University of California, San Diego, CA 92093, USA; bcortez@health.ucsd.edu; 2 School of Public Health, San Diego State University, San Diego, CA 92182, USA; tianying.wu@sdsu.edu; 3 Department of Environmental Health Sciences, Mailman School of Public Health, Columbia University, New York, NY 10027, USA; rps1@cumc.columbia.edu; 4 Department of Medicine, Vagelos College of Physicians and Surgeons and Department of Epidemiology, Mailman School of Public Health, Columbia University, New York, NY 10027, USA; ain1@cumc.columbia.edu; 5 Department of Environmental Medicine and Public Health, Icahn School of Medicine at Mount Sinai, New York, NY 10029, USA; mary.wolff@mssm.edu (M.S.W.); jia.chen@mssm.edu (J.C.); susan.teitelbaum@mssm.edu (S.L.T.); 6 UC San Diego Moores Cancer Center, San Diego, CA 92037, USA; 7 Department of Radiation Medicine & Applied Science, University of California, San Diego, CA 92037, USA

**Keywords:** dietary acid load, potential renal acid load, net endogenous acid production, breast cancer, mortality, survival, polychlorinated biphenyls

## Abstract

Dietary acid load (DAL) may be associated with all-cause mortality (ACM) and breast cancer-specific mortality (BCM), and these associations may be modified by serum polychlorinated biphenyl (PCB) levels. Participants included 519 women diagnosed with first primary in situ or invasive breast cancer in 1996/1997 with available lipid-corrected PCB data. After a median of 17 years, there were 217 deaths (73 BCM). Potential renal acid load (PRAL) and net endogenous acid production (NEAP) scores calculated from a baseline food frequency questionnaire estimated DAL. Cox regression estimated covariate-adjusted hazard ratios (HRs) and 95% confidence intervals (CIs) for associations between PRAL and NEAP with mortality. We evaluated effect measure modification by total serum PCB levels (>median vs. ≤median). PRAL quartile 4 versus quartile 1 was associated with an ACM HR of 1.31 (95%CI = 0.90–1.92). In the upper median of PCBs, ACM HRs were 1.43 (95%CI = 0.96–2.11) and 1.40 (95%CI = 0.94–2.07) for PRAL and NEAP upper medians, respectively. In the lower median of PCBs, the upper median of NEAP was inversely associated with BCM (HR = 0.40, 95%CI = 0.19–0.85). DAL may be associated with increased risk of all-cause mortality following breast cancer among women with high total serum PCB levels, but inversely associated with breast cancer mortality among women with low PCB levels.

## 1. Introduction

In 2012, the American Cancer Society (ACS) published its nutrition and physical activity guidelines, which included recommendations of balancing caloric intake with physical activity, eating five or more vegetable and fruit servings each day, and limiting the consumption of processed and red meat [1]. Since publication of the ACS guidelines, numerous observational epidemiologic studies and randomized clinical trials (RCTs) have been conducted to elucidate the role of diet on breast cancer risk and to refine dietary recommendations for women with breast cancer. In these studies, higher adherence to the Chinese Food Pagoda and to the Dietary Approaches to Stop Hypertension diets was reported to be associated with reduced risk of overall mortality and recurrence among breast cancer survivors [2]. In the Women’s Intervention Nutrition Study RCT, reducing fat intake was associated with improvements in relapse-free survival in post-menopausal women with breast cancer [3]. These diets, characterized by high intake of fruits and vegetables and low intake of meat, suggest that improvements in breast cancer prognosis may occur through influences on the body’s acid–base balance.

The normal function of the body’s physiological processes is tied to proper acid–base balance [4]. As such, even slight variations in the acid–base ratio may result in changes in oxygen delivery to tissues, structure of proteins, and biochemical properties [5]. Of relevance to breast cancer, an acid–base imbalance may also result in increased inflammation [6,7] and alterations in the tissue microenvironment [8,9,10]. Diet is a major determinant of acid load and is influenced by the intake of acid-inducing (e.g., meats and cheeses) and base-inducing (e.g., fruits and vegetables) foods [7]. As such, dietary acid load (DAL) is estimated in epidemiologic studies using food frequency questionnaires (FFQs) [11]. Results from studies of the association between FFQ-estimated DAL and risk of incident breast cancer have been mixed [12,13,14,15] and, to date, only one study has examined DAL in association with mortality following breast cancer [16]. In that study, the rates of all-cause and breast cancer mortality were elevated 54% and 52%, respectively, for the highest versus the lowest quartiles of NEAP, and associations were stronger among smokers with over 15 pack-years [16].

Using resources from the Long Island Breast Cancer Study Project (LIBCSP), a population-based study of breast cancer, we examined whether DAL was associated with increased risk of long-term mortality following breast cancer. We also examined whether this association was modified by total levels of serum polychlorinated biphenyls (PCBs), synthetic, organic and toxic compounds given that the kidneys serve the important role of maintaining acid–base balance of the body [17,18] and PCBs have been associated with nephropathy [19] and are hypothesized to increase the risk of breast cancer mortality [20,21]. We hypothesized that higher DAL would be associated with a higher risk of mortality following breast cancer and that this association would be more pronounced among women with higher PCB levels than among women with lower PCB levels.

## 2. Materials and Methods

### 2.1. Study Design

The LIBCSP is a population-based study of breast cancer initiated as a case–control study with subsequent follow-up of participants to identify breast cancer prognostic factors [22,23]. Breast cancer cases were women from the Nassau and Suffolk Counties on Long Island, New York with newly diagnosed first primary in situ or invasive breast cancer between 1 August 1996 and 31 July 1997 [22]. Among the 1508 women with breast cancer who completed the in-person interview, on average, within three months of breast cancer diagnosis, we excluded 27 women without FFQ data and 890 women without data on lipid-adjusted PCBs as described below, resulting in an analytic sample size of 591. Institutional Review Board approval was obtained from all participating institutions and written informed consent was obtained prior to study participation.

### 2.2. Outcome Assessment

The 591 women with breast cancer included in this study were followed from enrollment in 1996/1997 through 31 December 2014. Date and causes of death were determined by linkage to the National Death Index. Breast cancer-related deaths were identified using the International Statistical Classification of Diseases codes 174.9 and C-50.9 listed on the death certificate. For analyses of all-cause mortality (ACM), women alive at the end of follow-up were censored. For analyses of breast cancer-specific mortality (BCM), we also censored women who died from other causes at the time of death. After a median follow-up of 17 years (range = 0.41–18.41 years), we identified 217 deaths, 73 of which were due to breast cancer.

### 2.3. Dietary Assessment

All 1508 LIBCSP women with breast cancer completed a comprehensive interviewer-administered questionnaire on known and suspected breast cancer risk factors. Of these 1481 also completed a modified Block FFQ that captured intake, usual frequency, and portion sizes of ~100 foods and beverages in the 12 months before diagnosis [24]. The FFQ items included eight frequency options for each food item, from <1 per month to 2+ per day. Reference average servings were provided, and participants indicated whether their portion consumption was less, average, or more than the indicated amount [24,25]. Daily nutrient intakes were estimated by converting frequencies and portion sizes using the National Cancer Institute’s DietSys Version 3 [26]. These nutrient values were used to estimate DAL by calculating the potential renal acid load [PRAL, [27]] and net endogenous acid production [NEAP, [28]] scores using the following formulas:

PRAL (mEq/day) = (0.49 × protein (g/day)) + (0.037×phosphorus (mg/day)) − (0.021 × potassium (mg/day)) − (0.026 × magnesium (mg/day)) − (0.013 × calcium (mg/day))

NEAP (mEq/day) = (54.5 × protein (g/day)/potassium (mEq/day)) − 10.2.

PRAL measures the renal net acid excretion by measuring the mean intestinal absorption rates for individual nutrients and post-absorptive metabolism of sulfur-containing amino acids [11]. NEAP facilitates the measurement of diet-dependent acidosis by focusing on protein and potassium contents of an individual’s diet due to their role in the production of sulfuric acid and bicarbonate following consumption [28]. In analyses, we used total calorie-adjusted PRAL and NEAP scores, which were derived by adding the residuals from generalized linear models regressing PRAL/NEAP on total caloric intake to the sample means of PRAL (mean = 1.69 mEq/day) and NEAP (mean = 44.55 mEq/day) [29].

### 2.4. PCBs Measurement

At the time of the interview, 1102 of the 1508 women with breast cancer provided a non-fasting 40 mL blood sample for laboratory analyses, including the quantification of 29 PCB congeners and total lipids in a subsample (*n* = 646), as previously reported [23]. Samples selected for laboratory analyses included a random sample from women with invasive breast cancer (*n* = 415), all samples from women with in situ tumors and from those initially categorized as in situ that were subsequently determined to be invasive (*n* = 226), and samples from African American women who were not selected in the first three steps (*n* = 5). Of the women who provided blood samples, 77% were collected prior to chemotherapy initiation [30]. PCBs were measured by gas chromatography and electron capture detection using the method described by Brock et al. [31]. PCB levels below the limit of detection (LOD = 0.07 ng/mL) were set to 0 ng/mL. Total PCBs was computed as the sum of the 29 lipid-adjusted PCBs and dichotomized at the median.

### 2.5. Covariates

Covariates were identified a priori based on previous studies of DAL and breast cancer incidence/mortality [6,16,32], and using directed acyclic graphs. The covariates were included in our multivariable regression models to control for confounding of the association between DAL and mortality following breast cancer. Model 1 covariates included age at diagnosis (continuous in years). Model 2 covariates included age, education (<high school-high school graduate [ref]; some college or college graduate; or post-college), menopausal status (pre-menopausal [ref] or post-menopausal), income (≤$24,999 United States Dollars [USD] [ref]; $25,000 to ≤$49,999 USD; or ≥$50,000 USD), body mass index (BMI, continuous in kg/m^2^), parity and lactation (nulliparous [ref]; parous/never lactated; or parous/ever lactated), alcohol intake (non-drinkers [ref]; lifetime intake less than 15 g/day; lifetime intake between 15 and 30 g/day; lifetime intake ≥30 g/day), smoking status (never smoker [ref]; current smoker within the last 12 months; or former smoker), and breast cancer stage at diagnosis (in situ [ref] or invasive). We also included adjustment for PCBs (≤median [ref] vs. >median), as appropriate.

### 2.6. Statistical Analysis

We used Cox regression modeling to estimate hazard ratios (HRs) and 95% confidence intervals (CIs) of the associations between PRAL and NEAP with ACM and BCSM from diagnosis in 1996/1996 to 31 December 2014. We examined quartiles of PRAL and NEAP scores (reference = Quartile 1) as well as the continuous scores per standard deviation (SD) increases. The *P*_Trend_ was the *p*-value of the continuous PRAL or NEAP (per SD) variables in age-adjusted and fully adjusted cox regression models. The proportional hazards assumption was assessed using Schoenfeld residuals, which were independent of time [i.e., time, log(time), or time^2^ (time × time)]. In sensitivity analyses, we restricted the follow-up to seven years to examine whether DAL (using median splits of PRAL and NEAP) was associated with short-term mortality following breast cancer.

To examine whether total serum PCB levels modified the associations between PRAL and NEAP with ACM and BCSM, effect measure modification on the multiplicative scale was evaluated by conducting stratified analyses (total PCBs >median versus ≤median) and by including median split DAL-by-dichotomous PCB level interactions in the Cox regression models. These associations were visualized using forest plots for the fully adjusted models. Interaction on the additive scale was evaluated by examining joint indicators of PRAL or NEAP and PCB levels (i.e., low PRAL or NEAP/low PCB levels (reference); high/low; low/high; high/high) in the fully adjusted model, which were used to compute interaction contrast ratios (ICRs) and 95% CIs [33]. The absence of interaction on the additive scale is indicated by an ICR value of 0.

## 3. Results

Of the 591 women included in this study, 72.1% were 50 years or older, 44.2% had high school or less education, 51.1% had an annual income of $50,000 or greater, and 20.8% had a BMI ≥ 30 kg/m^2^ (Table 1). The median levels of total calorie-adjusted DAL scores were 2.14 (Interquartile Range [IQR] = −3.53–7.20) mEq/day for PRAL and 43.4 (IQR = 36.61–51.05) mEq/Day for NEAP. Compared to women in the upper median of PRAL, women in the lower median had higher proportions of women ages 50 or older (79.8% vs. 63.6%), with some college or graduate education or greater (58.8% vs. 52.6%), were former smokers (41.2% versus 27.9%) and were post-menopausal (71.8% vs. 59.4%). Similar patterns were seen by NEAP median splits.

The associations between DAL with ACM and BCSM are reported in Table 2. In Model 1, the highest (vs. lowest) quartiles of PRAL were associated with HRs of 1.20 (95%CI = 0.95–1.51) for ACM and 0.95 (95%CI = 0.66–1.36) for BCSM. HR estimates were more pronounced in fully adjusted models (Model 2) with HRs of 1.31 (95%CI = 0.90–1.23) for ACM and 0.89 (95%CI = 0.46–1.72) for BCSM. For NEAP, the highest (vs. lowest) quartiles of NEAP were associated with HRs of 1.09 (95%CI = 0.87–1.37) for ACM and 1.00 (95%CI = 0.69–1.45) for BCSM in Model 1. The HR was similar in Model 2 for ACM (HR = 1.07, 95%CI = 0.74–1.55), but inverse for BCSM (HR = 0.78, 95%CI = 0.40–1.51) (Table 2). In sensitivity analyses examining 7-year mortality among all women overall, BCSM rates were increased in the upper (vs. lower) medians of PRAL (HR = 1.30 95%CI = 0.81–2.08) and NEAP (HR = 1.24 95%CI = 0.77–1.99) (Table 1).

The associations between joint indictors of DAL and total PCB levels are reported in Table 3. Based on the ICR, there was excess risk associated with NEAP in Models 1 (ICR = 0.47 95%CI = 0.03, 0.90) and 2 (ICR = 0.55 95%CI = 0.12, 0.97) for ACM; and in Models 1 (ICR = 0.86 95%CI = 0.28, 1.43) and 2 (ICR = 0.68 95%CI: 0.09, 1.27) for BCSM. There was excess risk of BCSM for PRAL in Models 1 (ICR = 0.77 95%CI = 0.13, 1.41) and 2 (ICR = 0.82 95%CI = 0.18, 1.46) (Table 3). 

**Figure 1:**
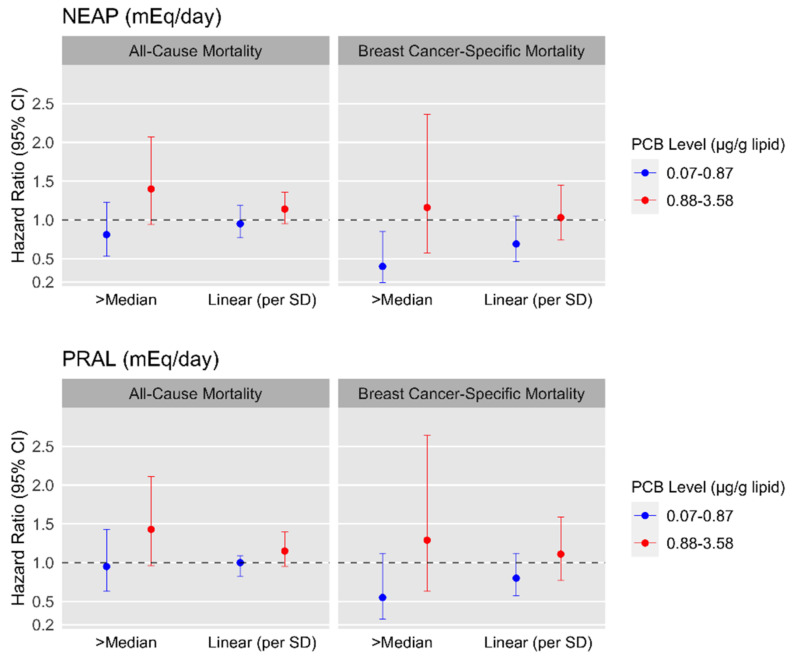
Forest plot of hazard ratios (HRs) and 95% confidence intervals (CIs) for the fully adjusted associations between dietary acid load scores and all-cause and breast cancer-specific mortality by serum total lipid-adjusted PCB levels (*n* = 591). Model is adjusted for age at diagnosis (continuous in years), education (<high school-high school graduate [ref]; somecollege or college graduate; or post-college), menopausal status (pre-menopausal [ref] or post-menopausal), income (≤$24,999 United States Dollars [USD]; $25,000 to ≤$49,999 USD; or ≥$50,000 USD), body mass index (BMI, continuous in kg/m^2^), parity and lactation (nulliparous [ref]; parous/never lactated; or parous/ever lactated), alcohol intake (non-drinkers [ref]; lifetime intake less than 15 g/day; lifetime intake between 15 and 30 g/day; lifetime intake ≥30 g/day), smoking status (never smoker [ref]; current smoker within the last 12 months; or former smoker), and breast cancer stage at diagnosis (in situ [ref] or invasive).

The associations between DAL with ACM and BCSM stratified by PCB levels are reported in Table 4, and the fully adjusted associations are visualized in Figure 1. In the upper median of PCB levels, a one-SD increase in PRAL was associated with an ACM HR of 1.15 (95%CI = 0.95–1.40) in Model 2 (Table 4). For ACM, *p*-values for the multiplicative interactions (*P*_Interaction_) between NEAP/PCBs and PRAL/PCBs in fully-adjusted models were 0.02 and 0.06, respectively. For BCSM, all NEAP or PRAL-by-PCB *P*_Interaction_’s were ≥0.73. In the upper median of PCBs, mortality rates were elevated for ACM for the upper (vs. lower) median of PRAL (HR = 1.43, 95%CI = 0.96–2.11) and NEAP (HR = 1.40, 95%CI = 0.94–2.07) in Model 2. In the lower median of PCBs for Model 2, the upper (vs. lower) median of NEAP was inversely associated with BCSM (HR = 0.40, 95%CI = 0.19–0.85); this association was null in the upper median of PCBs (HR = 1.16, 95%CI = 0.57–2.36) (Table 4). In sensitivity analyses in which we examined 7-year mortality overall and stratified by PCB levels. no associations were observed between for the overall model (Table 1). There were elevated ACM rates among women in the upper PCB medians with upper (vs. lower) median of NEAP (HR = 2.03 95%CI = 1.04–3.97) and PRAL (HR = 2.21 95%CI = 1.12–4.33) in Model 2 (Table 4). The *p*-values for multiplicative interaction terms were ≤0.10 for all NEAP or PRAL-by-PCB interactions.

## 4. Discussion

In this study of mortality following breast cancer, DAL at diagnosis was not associated with increased risk of ACM or BCSM after a median 17 years of follow-up among all women. However, PCB exposure modified the relationship between DAL and ACM; we identified interactions between PCBs and DAL on the additive and multiplicative scales. In the lower median of PCB levels, higher NEAP scores were inversely associated with mortality, while in the upper median of PCB levels, higher DAL was associated with an increased risk of ACM.

To date, the relationship between DAL and mortality among women with breast cancer has only been examined in one study [16]. In the WHEL Study, higher NEAP and PRAL scores were associated with increased rates of ACM and BCSM [16]. The differences between our results may be due to several reasons. First, the WHEL study recruited breast cancer survivors who were diagnosed 2–4 years before enrollment, while the LIBCSP enrolled women within 3 months of diagnosis. Second, the WHEL study measured dietary intakes using a 24 h dietary recall, while our study used a FFQ that assessed pre-diagnosis diet. Third, given diet changes throughout the course of cancer treatment [34], longitudinal measures of dietary intake may better recover the association than only a baseline measure. The PRAL and NEAP scores reported in the WHEL Study were also more alkaline than those reported in the LIBCSP [16]. Last, the follow-up periods differed between the WHEL study (7 years) and LIBCSP (17 years). Although we did not observe a strong association between baseline DAL and 7-year mortality overall, when stratifying by PCB associations between NEAP and PRAL with mortality were strengthened in the upper median of PCB exposure. Therefore, the interaction effect between PCB exposure and DAL is more evident at a shorter follow-up period.

Several hypotheses underlying the association between DAL and breast cancer mortality have been proposed. Cancer patients and survivors have been reported to have reduced kidney function, which can lead to increased susceptibility to higher acidogenic diets [35,36,37,38]; acute renal failure and chronic renal dysfunction have also been reported as a consequence of breast cancer treatments including chemotherapy and may persist following the completion of treatment [39,40]. Injured kidneys are less efficient at stabilizing the acid–base balance and can lead to metabolic acidosis [41,42], which has been linked to increased invasiveness of breast cancer cells [8,10]. Thus, breast cancer survivors are at an increased risk of metabolic acidosis, which can be augmented by a highly acidic diet. On the other hand, the inverse association we observed between DAL and breast cancer mortality among women with low levels of PCBs requires further investigation. At least one recent study suggests that DAL may have a non-linear association with health outcomes [43].

The mechanism of action by which DAL impacts overall mortality may also be due to its association with other comorbidities. The association between PRAL and ACM may be related to the impact of diet’s acidity on the cardiovascular system, as excess diet alkalinity and acidity was associated with higher mortality from cardiovascular conditions in Swedish and Japanese adults [44,45]. High PRAL scores have also been associated with increased risk for depression in breast cancer survivors, a condition that is found to reduce quality of life and increase ACM risk [46,47]. Last, metabolic acidosis has also been found to impair the immune response and lead to muscle degradation [48].

The additive effects of PCB exposure and acidogenic diets on mortality of cancer survivors has not been well established, but may be due to the negative effects of PCBs on kidney function. The kidneys are crucial to maintaining acid–base balance, as the metabolic process which generates both volatile (CO_2_ respiration) and non-volatile (H+ excretion) acid from consumed food are excreted by this organ [7,49]. Prior studies have reported that exposure to PCBs 153 and 77 reduces the proper function of kidney and liver cells. In in vitro studies, PCB exposure induced and accelerated apoptotic death of kidney cells [50]. A mass PCB food poisoning of 1900 individuals in Japan resulted in multiple health effects, including high uric acid concentrations, suggesting that exposure contributed to acid–base imbalance [19,51]. Given that both PCB exposure and cancer may affect renal function, the joint contributions may explain our findings reported here.

The strengths of this study included the prospective cohort design with long-term follow-up among a population-based sample of women, and passive linkage to the NDI, which allowed near complete ascertainment of deaths. However, several limitations should be noted. First, we only considered one pre-diagnosis measurement of DAL and had only one measurement of PCB levels from blood samples collected at diagnosis, and thus we were unable to account for changes in diet or PCB levels over time. Second, we relied on a subjective measure of diet (i.e., FFQ), which may have higher measurement errors in assessing DAL than, for example, repeated 24 h recalls and we did not use a biomarker of DAL to confirm pH levels estimated using PRAL and NEAP values. Third, we did not collect information on metastasis in the LIBCSP; however, breast cancer stage at diagnosis was included in the models to help control for confounding by severity of breast cancer. Last, the LIBCSP was comprised primarily of White women and therefore the findings might not be generalizable to the all women with breast cancer. Future studies should consider collecting multiple biomarker-based measurements of DAL over time in a diverse sample of women with breast cancer, to better understand the relationship between DAL and mortality.

## 5. Conclusions

Although we did not find a strong relationship between baseline DAL and mortality following breast cancer, there was a modest increase in risk of ACM among women with higher PCB levels and higher DAL. Women with breast cancer with diets high in animal fats, thus, may be at increased risk of mortality due to the resulting acid–base imbalance and exposure to PCBs. Although exposure to PCBs has declined since their use and production have been banned in the US, some PCB congeners are still detectable at high levels in US children and adults [52]. This is coupled with the fact there has been a rise in acidic diets in the Western world, as consumption of dairy and meat products have grown with a reduction in alkaline foods. Therefore, gaining better understanding of its potential role on mortality is crucial to help clinicians provide proper dietary guidelines for their patients in order to improve long-term survival of breast cancer survivors.

## Figures and Tables

**Table 1:** Characteristics of the Long Island Breast Cancer Study Project women with breast cancer (*n* = 591).

Characteristics	Overall	PRAL		NEAP	
		≤2.14 mEq/day	>2.14 mEq/day	≤43.4 mEq/day	>43.4 mEq/day
	*n* (%)	*n* (%)	*n* (%)	*n* (%)	*n* (%)
*n*	591	308	283	306	285
**Age at Diagnosis (years)**					
<50	165 (27.9%)	62 (20.1%)	103 (36.4%)	59 (19.3%)	106 (37.2%)
50–64	234 (39.6%)	135 (43.8%)	99 (35.0%)	131 (42.8%)	103 (36.1%)
≥65	192 (32.5%)	111 (36.0%)	81 (28.6%)	116 (37.9%)	76 (26.7%)
Mean (SD)	58.1 (12.1)	60.0 (11.3)	56.1 (12.6)	60.5 (11.4)	55.6 (12.4)
**Education (USD)**					
<HS-HS graduate	261 (44.2%)	127 (41.2%)	134 (47.3%)	130 (42.5%)	131 (46.0%)
Some college/graduate	236 (39.9%)	134 (43.5%)	102 (36.0%)	128 (41.8%)	108 (37.9%)
Post-college	94 (15.9%)	47 (15.3%)	47 (16.6%)	48 (15.7%)	46 (16.1%)
**Annual Income**					
<$24,999	113 (19.1%)	54 (17.5%)	59 (20.8%)	59 (19.3%)	54 (18.9%)
$25,000–$49,999	176 (29.8%)	103 (33.4%)	73 (25.8%)	101 (33.0%)	75 (26.3%)
≥$50,000	302 (51.1%)	151 (49.0%)	151 (53.4%)	146 (47.7%)	156 (54.7%)
**BMI (kg/m^2^)**					
<25	269 (45.5%)	137 (44.5%)	132 (46.6%)	140 (45.8%)	129 (45.3%)
25–29	199 (33.7%)	114 (37.0%)	85 (30.0%)	110 (35.9%)	89 (31.2%)
≥30	123 (20.8%)	57 (18.5%)	66 (23.3%)	56 (18.3%)	67 (23.5%)
Mean (SD)	26.5 (5.5)	26.4 (5.1)	26.7 (6.0)	26.3 (5.0)	26.8 (6.1)
**Menopausal Status**					
Pre-menopausal	202 (34.2%)	87 (28.2%)	115 (40.6%)	80 (26.1%)	122 (42.8%)
Post-menopausal	389 (65.8%)	221 (71.8%)	168 (59.4%)	226 (73.9%)	163 (57.2%)
**Parity and Lactation History**					
Nulliparous	78 (13.2%)	38 (12.3%)	40 (14.1%)	36 (11.8%)	42 (14.7%)
Parous/Never lactated	310 (52.5%)	155 (50.3%)	155 (54.8%)	159 (52.0%)	151 (53.0%)
Parous/Ever lactated	203 (34.3%)	115 (37.3%)	88 (31.1%)	111 (36.3%)	92 (32.3%)
**Lifetime Alcohol Intake (g/day)**					
Non-drinkers	217 (36.7%)	110 (35.7%)	107 (37.8%)	113 (36.9%)	104 (36.5%)
<15	288 (48.7%)	148 (48.1%)	140 (49.5%)	144 (47.1%)	144 (50.5%)
15–30	60 (10.2%)	34 (11.0%)	26 (9.2%)	32 (10.5%)	28 (9.8%)
≥30	26 (4.4%)	16 (5.2%)	10 (3.5%)	17 (5.6%)	9 (3.2%)
**Smoking Status**					
Never smoker	283 (47.9%)	140 (45.5%)	143 (50.5%)	142 (46.4%)	141 (49.5%)
Current smoker	102 (17.3%)	41 (13.3%)	61 (21.6%)	41 (13.4%)	61 (21.4%)
Former smoker	206 (34.9%)	127 (41.2%)	79 (27.9%)	123 (40.2%)	83 (29.1%)
**Stage Status**					
In situ	169 (28.6%)	84 (27.3%)	85 (30.0%)	84 (27.5%)	85 (29.8%)
Invasive	422 (71.4%)	224 (72.7%)	198 (70.0%)	222 (72.5%)	200 (70.2%)
**Nodal Involvement**					
No	255 (43.1%)	128 (41.6%)	127 (44.9%)	131 (42.8%)	124 (43.5%)
Yes	113 (19.1%)	62 (20.1%)	51 (18.0%)	62 (20.3%)	51 (17.9%)
Missing	255	128	127	131	124
**ER/PR Status**					
ER−/PR−	261 (44.2%)	138 (44.8%)	123 (43.5%)	142 (46.4%)	119 (41.8%)
ER−/PR+	73 (12.4%)	33 (10.7%)	40 (14.1%)	33 (10.8%)	40 (14.0%)
ER+/PR−	73 (12.4%)	33 (10.7%)	40 (14.1%)	33 (10.8%)	40 (14.0%)
ER+/PR+	41 (6.9%)	27 (8.8%)	14 (4.9%)	26 (8.5%)	15 (5.3%)
Missing	261	138	123	142	119
**Vital Status**					
Alive	374 (63.3%)	195 (63.3%)	179 (63.3%)	189 (61.8%)	185 (64.9%)
Deceased	217 (36.7%)	113 (36.7%)	104 (36.7%)	117 (38.2%)	100 (35.1%)
BRCA death	73 (12.4%)	39 (12.7%)	34 (12.0%)	41 (13.4%)	32 (11.2%)

BMI, body mass index; ER, estrogen receptor; g, grams; HS, high school; kg, kilograms; m, meter; mEq, milliequivalent; NEAP, net endogenous acid production; PRAL, potential renal acid load; PR, progesterone receptor; SD, standard deviation; USD, United States dollar.

**Table 2:** Hazard ratios (HRs) and 95% confidence intervals (CIs) for the associations between dietary acid load and all-cause and breast cancer-specific mortality in the Long Island Breast Cancer Study Project (*n* = 591).

Dietary Acid Load	All-Cause Mortality					
			Model 1 ^a^		Model 2 ^b^	
	Deaths	Censored	HR (95%CI)	*P*_Trend_^c^	HR (95%CI)	*P*_Trend_^c^
**PRAL (mEq/day)**						
−46.5 to −3.5 (Q1)	57	101	1.00 (Ref.)		1.00 (Ref.)	
−3.6 to 2.1	56	94	1.04 (0.83–1.31)		1.19 (0.81–1.75)	
2.2 to 7.2	46	84	1.12 (0.89–1.40)		1.16 (0.78–1.72)	
7.3 to 38.9 (Q4)	58	95	1.20 (0.95–1.51)		1.31 (0.90–1.92)	
Linear (per SD)	217	374	1.10 (0.96–1.26)	0.19	1.08 (0.93–1.23)	0.34
**NEAP (mEq/day)**						
11.8 to 36.5 (Q1)	65	97	1.00 (Ref.)		1.00 (Ref.)	
36.6 to 43.4	52	92	0.97 (0.77–1.22)		0.96 (0.66–1.40)	
43.5 to 51.0	44	90	1.04 (0.83–1.30)		1.02 (0.68–1.52)	
51.1 to 112.8 (Q4)	56	95	1.09 (0.87–1.37)		1.07 (0.74–1.55)	
Linear (per SD)	217	374	1.10 (0.96–1.25)	0.14	1.06 (0.92–1.21)	0.42
**Dietary Acid Load**	**Breast Cancer-Specific Mortality**					
			**Model 1 ^a^**		**Model 2 ^b^**	
	**Deaths**	**Censored**	**HR (95%CI)**	***P*_Trend_^c^**	**HR (95%CI)**	***P*_Trend_^c^**
**PRAL (mEq/day)**						
−46.5 to ≤ −3.5 (Q1)	21	137	1.00 (Ref.)		1.00 (Ref.)	
−3.5 to ≤2.14	18	132	0.78 (0.54–1.14)		0.96 (0.50–1.84)	
2.1 to ≤7.2	15	115	0.93 (0.65–1.33)		0.81 (0.41–1.62)	
7.2 to ≤38.9 (Q4)	19	134	0.95 (0.66–1.36)		0.89 (0.46–1.72)	
Linear (per SD)	73	518	0.98 (0.77–1.24)	0.49	0.96 (0.75–1.23)	0.73
**NEAP (mEq/day)**						
11.8 to ≤36.5 (Q1)	22	140	1.00 (Ref.)		1.00 (Ref.)	
36.6 to ≤43.4	19	125	0.96 (0.66–1.38)		0.96 (0.51–1.81)	
43.4 to ≤51.0	14	120	1.00 (0.69–1.44)		0.66 (0.32–1.34)	
51.1 to ≤112.8 (Q4)	18	133	1.00 (0.69–1.45)		0.78 (0.40–1.51)	
Linear (per SD)	73	518	0.96 (0.75–1.22)	0.58	0.91 (0.71–1.18)	0.49

Long Island Breast Cancer Study Project (LIBCSP) participants diagnosed with breast cancer between 1 August 1996 and 31 July 1997 and followed-up through 31 December 2014. CI, confidence interval; HR, hazard ratio; mEq, milliequivalent; NEAP, net endogenous acid production; PRAL, potential renal acid load; Ref., reference; SD, standard deviation; ^a^ Model 1 is adjusted for age at diagnosis (continuous in years). ^b^ Model 2 is adjusted for age at diagnosis (continuous in years), education (<high school-high school graduate [ref]; some college or college graduate; or post-college), menopausal status (pre-menopausal [ref] or post-menopausal), income (≤$24,999 United States Dollars [USD]; $25,000 to ≤$49,999 USD; or ≥$50,000 USD), body mass index (BMI, continuous in kg/m^2^), parity and lactation (nulliparous [ref]; parous/never lactated; or parous/ever lactated), alcohol intake (non-drinkers [ref]; lifetime intake less than 15 g/day; lifetime intake between 15 and 30 g/day; lifetime intake ≥30 g/day), smoking status (never smoker [ref]; current smoker within the last 12 months; or former smoker), breast cancer stage at diagnosis (in situ [ref] or invasive), and PCB level (lower median [ref] or upper median); ^c^ P_Trend_ is the *p*-value from Cox regression models using continuous measures of DAL.

**Table 3:** Hazard ratios (HR) and 95% confidence intervals (CIs) for the associations between the joint effects of dietary acid load and total serum PCB levels and all-cause and breast cancer-specific mortality in the Long Island Breast Cancer Study Project (*n* = 591).

		All-Cause Mortality			
		Model 1		Model 2	
Dietary Acid Load	PCB Levels	HR (95%CI) ^a^	ICR (95%CI) ^a^	HR (95%CI) ^b^	ICR (95%CI) ^b^
**NEAP (mEq/day)**	**PCB (μg/g lipid)**				
11.8 to 43.4 (≤med)	0.07 to 0.87 (≤med)	1.00 (Ref.)	-	1.00 (Ref.)	-
43.5 to 112.8 (>med)	0.07 to 0.87 (≤med)	0.89 (0.60–1.32)	-	0.78 (0.52–1.16)	-
11.8 to 43.4 (≤med)	0.87 to 3.58 (>med)	0.69 (0.48–1.00)	-	0.74 (0.50–1.08)	-
43.5 to 112.8 (>med)	0.87 to 3.58 (>med)	1.04 (0.72–1.52)	0.47 (0.03, 0.90)	1.04 (0.71–1.52)	0.55 (0.12, 0.97)
**PRAL (mEq/day)**	**PCB (μg/g lipid)**				
−46.45 to 2.1 (≤med)	0.07 to 0.87 (≤med)	1.00. (Ref.)	-	1.00. (Ref.)	-
2.2 to 38.9 (>med)	0.07 to 0.87 (≤med)	1.03 (0.70–1.53)	-	0.90 (0.61–1.35)	-
−46.45 to 2.1 (≤med)	0.87 to 3.58 (>med)	0.74 (0.51–1.09)	-	0.79 (0.53–1.16)	-
2.2 to 38.9 (>med)	0.87 to 3.58 (>med)	1.10 (0.75–1.61)	0.33 (−0.15, 0.82)	1.11 (0.75–1.63)	0.46 (−0.01, 0.92)
		**Breast Cancer-Specific Mortality**			
		**Model 1**		**Model 2**	
**Dietary Acid Load**	**PCB Levels**	**HR (95%CI) ^a^**	**ICR (95%CI) ^a^**	**HR (95%CI) ^a^**	**ICR (95%CI) ^a^**
NEAP (mEq/day)	PCB (μg/g lipid)				
11.8 to 43.4 (≤med)	0.07 to 0.87 (≤med)	1.00 (Ref.)	-	1.00 (Ref.)	-
43.5 to 112.8 (>med)	0.07 to 0.87 (≤med)	0.44 (0.22–0.89)	-	0.40 (0.20–0.83)	-
11.8 to 43.4 (≤med)	0.87 to 3.58 (>med)	0.63 (0.33–1.18)	-	0.66 (0.34–1.26)	-
43.5 to 112.8 (>med)	0.87 to 3.58 (>med)	0.92 (0.51–1.67)	0.86 (0.28, 1.43)	0.86 (0.47–1.59)	0.68 (0.09, 1.27)
**PRAL (mEq/day)**	**PCB (μg/g lipid)**				
−46.45 to 2.1 (≤med)	0.07 to 0.87 (≤med)	1.00 (Ref.)	-	1.00 (Ref.)	-
2.2 to 38.9 (>med)	0.07 to 0.87 (≤med)	0.58 (0.30–1.14)	-	0.54 (0.27–1.07)	-
−46.45 to 2.1 (≤med)	0.87 to 3.58 (>med)	0.68 (0.36–1.30)	-	0.69 (0.36–1.34)	-
2.2 to 38.9 (>med)	0.87 to 3.58 (>med)	1.04 (0.56–1.90)	0.77 (0.13, 1.41)	0.99 (0.53–1.86)	0.82 (0.18, 1.46)

Long Island Breast Cancer Study Project (LIBCSP) participants diagnosed with breast cancer between 1 August 1996 and 31 July 1997 and followed-up through 31 December 2014. CI, confidence interval; HR, hazard ratio; mEq, milliequivalent; NEAP, net endogenous acid production; PRAL, potential renal acid load; Ref., reference. ^a^ Model 1 is adjusted for age at diagnosis (continuous in years). ^b^ Model 2 is adjusted for age at diagnosis (continuous in years), education (<high school-high school graduate [ref]; some college or college graduate; or post-college), menopausal status (pre-menopausal [ref] or post-menopausal), income (≤$24,999 United States Dollars [USD]; $25,000 to ≤$49,999 USD; or ≥$50,000 USD), body mass index (BMI, continuous in kg/m^2^), parity and lactation (nulliparous [ref]; parous/never lactated; or parous/ever lactated), alcohol intake (non-drinkers [ref]; lifetime intake less than 15 g/day; lifetime intake between 15 and 30 g/day; lifetime intake ≥30 g/day), smoking status (never smoker [ref]; current smoker within the last 12 months; or former smoker), breast cancer stage at diagnosis (in situ [ref] or invasive), and lipid-adjusted PCB level (lower median [ref] or upper median).

**Table 4:** Hazard ratios (HRs) and 95% confidence intervals (CIs) for the associations between dietary acid load scores and all-cause and breast cancer-specific mortality by serum total lipid-adjusted PCB levels (*n* = 591).

	All-Cause Mortality								
	PCB Levels 0.07–0.87 μg/g Lipid (≤med)				PCB Levels 0.88–3.58 μg/g Lipid (>med)				
			Model 1 ^a^	Model 2 ^b^			Model 1 ^a^	Model 2 ^b^	
	**Deaths**	**Censored**	**HR (95%CI)**	**HR (95%CI)**	**Deaths**	**Censored**	**HR (95%CI)**	**HR (95%CI)**	***P*_Int._^c^**
**NEAP (mEq/day)**									0.02
11.8 to 43.4 (≤med)	45	105	1.00 (Ref.)	1.00 (Ref.)	55	80	1.00 (Ref.)	1.00 (Ref.)	
43.5 to 112.8 (>med)	55	91	0.90 (0.60–1.33)	0.81 (0.53–1.23)	62	98	1.50 (1.03–2.17)	1.40 (0.94–2.07)	
Linear (per SD)	100	196	0.96 (0.78–1.18)	0.95 (0.77–1.19)	117	178	1.19 (1.02–1.40)	1.14 (0.95–1.36)	
**PRAL (mEq/day)**									0.06
−46.45 to 2.1 (≤med)	52	94	1.00 (Ref.)	1.00 (Ref.)	61	101	1.00 (Ref.)	1.00 (Ref.)	
2.2 to 38.9 (>med)	48	102	1.04 (0.70–1.55)	0.95 (0.63–1.43)	56	77	1.47 (1.02–2.12)	1.43 (0.96–2.11)	
Linear (per SD)	100	196	1.00 (0.83–1.22)	1.00 (0.82–1.09)	117	178	1.19 (0.99–1.43)	1.15 (0.95–1.40)	
	**Breast Cancer-Specific Mortality**								
	**PCB Levels 0.07–0.87 μg/g Lipid (≤med)**				**PCB Levels 0.88–3.58 μg/g Lipid (>med)**				
			**Model 1 ^a^**	**Model 2 ^b^**			**Model 1 ^a^**	**Model 2 ^b^**	
	**Deaths**	**Censored**	**HR (95%CI)**	**HR (95%CI)**	**Deaths**	**Censored**	**HR (95%CI)**	**HR (95%CI)**	***P*_Int._^c^**
**NEAP (mEq/day)**									0.80
11.8 to 43.4 (≤med)	24	122	1.00 (Ref.)	1.00 (Ref.)	17	143	1.00 (Ref.)	1.00 (Ref.)	
43.5 to 112.8 (>med)	12	138	0.45 (0.23–0.92)	0.40 (0.19–0.85)	20	115	1.44 (0.74–2.80)	1.16 (0.57–2.36)	
Linear (per SD)	36	260	0.74 (0.51–1.08)	0.69 (0.46–1.05)	37	258	1.14 (0.86–1.51)	1.03 (0.74–1.45)	
**PRAL (mEq/day)**									0.73
−46.45 to 2.1 (≤med)	22	124	1.00 (Ref.)	1.00 (Ref.)	17	145	1.00 (Ref.)	1.00 (Ref.)	
2.2 to 38.9 (>med)	14	136	0.60 (0.30–1.19)	0.55 (0.27–1.12)	20	113	1.48 (0.77–2.85)	1.29 (0.63–2.64)	
Linear (per SD)	36	260	0.84 (0.61–1.15)	0.80 (0.57–1.12)	37	258	1.15 (0.83–1.60)	1.11 (0.77–1.59)	

Long Island Breast Cancer Study Project (LIBCSP) participants diagnosed with breast cancer between 1 August 1996 and 31 July 1997 and followed-up through 31 December 2014. CI, confidence interval; HR, hazard ratio; mEq, milliequivalent; NEAP, net endogenous acid production; PCB, polychlorinated biphenyls; PRAL, potential renal acid load; Ref., reference. ^a^ Model 1 is adjusted for age at diagnosis (continuous in years). ^b^ Model 2 is adjusted for age at diagnosis (continuous in years), education (<high school-high school graduate [ref]; some college or college graduate; or post-college), menopausal status (pre-menopausal [ref] or post-menopausal), income (≤$24,999 United States Dollars [USD]; $25,000 to ≤$49,999 USD; or ≥$50,000 USD), body mass index (BMI, continuous in kg/m^2^), parity and lactation (nulliparous [ref]; parous/never lactated; or parous/ever lactated), alcohol intake (non-drinkers [ref]; lifetime intake less than 15 g/day; lifetime intake between 15 and 30 g/day; lifetime intake ≥30 g/day), smoking status (never smoker [ref]; current smoker within the last 12 months; or former smoker), and breast cancer stage at diagnosis (in situ [ref] or invasive). ^c^ Pinteraction is the *p*-value from fully adjusted Cox regression models using median split DAL-by-dichotomous PCB level interactions.

## Data Availability

No appliable.
